# A Balamuthia survivor

**DOI:** 10.1099/jmmcr.0.005031

**Published:** 2016-06-28

**Authors:** Michael Eric Vollmer, Carol Glaser

**Affiliations:** ^1^​ Infectious Disease Doctors Medical Group, 365 Lennon Lane Suite 200, Walnut Creek, CA 94598, USA; ^2^​ The Permanente Medical Group, Oakland Medical Center, 3505 Broadway St, Oakland, CA 94611, USA

**Keywords:** balamuthia, ameba, encephalitis, headache, brain, survivor

## Abstract

**Introduction:**

This case report describes a human survivor of *Balamuthia mandrillaris* infection. This is a free-living amoeba that can cause infection with the devastating consequence of near universally fatal encephalitis. We report this case to demonstrate the possibility of recovery.

**Case presentation:**

A 26-year-old Hispanic male, a landscape gardener, presented to the hospital in March 2010 with a two month history of headache, visual disturbances and new-onset seizures. Brain imaging identified two enhancing central lesions and *Balamuthia mandrillaris* was later identified by brain biopsy. He received several months of various antimicrobials including miltefosine, a novel use of the drug in this disease at the time. Seven weeks into therapy, considerations were made to switch him to ‘comfort care’ because of worsening clinical status and seemingly lack of response to treatment. The patient finally demonstrated clinical and radiological improvement after eight weeks with modified therapy, despite experiencing some debilitating toxic effects likely to be related to antibiotics. Two years after his initial presentation he made a complete recovery.

**Conclusion:**

*Balamuthia mandrillaris* amoebic encephalitis is considered an almost universally fatal disease; this case demonstrates the possibility of recovery. This report outlines his treatment, drug toxicities and includes additional information regarding the therapeutic use of the drug miltefosine. Whether his survival is related to the specific antimicrobials used in this case is unknown and further investigation is warranted.

## Introduction

This case report describes a human survivor of *Balamuthia mandrillaris* infection. This is a free-living amoeba that can cause infection with the devastating consequence of near universally fatal encephalitis. *Balamuthia mandrillaris* was originally described in a pregnant mandrill monkey with encephalitis in 1986 and later identified as a human pathogen in 1990 (Visvesvara *et al.*, 1990). Since then, there have been approximately 200 reports worldwide – mostly from Latin America and the United States. The amoeba naturally inhabits soil and infection appears to occur via respiratory inhalation of aerosolized particles or direct inoculation through breaks in the skin (Deetz, 2003). Diagnosis is often delayed, and a vast majority of identified cases have resulted in death.

We report this case to demonstrate the possibility of recovery. This report outlines the patient’s treatment, drug toxicities and includes additional information regarding the therapeutic role of the drug miltefosine.

## Case report

The patient initially presented at an outside hospital emergency room (ER) in 2010 complaining of a few days of visual disturbances (e.g. floaters), light headedness, throbbing headache and syncope. Neuroimaging by computerized tomography (CT) was unremarkable. He was treated for dehydration and discharged. A follow-up magnetic resonance imaging (MRI) was recommended but not done at that time. He re-presented two days later with a generalized seizure to the same ER, started on phenytoin and was discharged.

Two months later, he presented to our institution with another generalized seizure and a history of persistent headache. Cerebrospinal fluid (CSF) analysis showed 2 leukocytes ×10^6^ l^−1^, 2 red blood cells ×10^6 ^l^−1^, protein 0.4 g l^−1^ , and glucose 3.4 mmol l^−1^. An MRI revealed two distinct cortical lesions: a 2.4 cm×2 cm×1.5 cm mass in the left frontal lobe and a 1.2 cm×1.6 cm×1.9 cm mass in the right temporal lobe. Both areas had a moderate to large amount of surrounding vasogenic edema. The patient was originally from Mexico and had been living in California for several years. He worked for an area landscaping company and lived in an urban zone without significant travel or exposure to the California Central Valley.

With this clinical history, he was initially treated empirically for neurocysticercosis with high dose dexamethasone and albendazole. These medications were discontinued approximately a week later when results of serological tests for cysticercosis [enzyme-linked immunosorbent assay (ELISA) IgG serum and CSF) returned negative from the laboratory, and the patient underwent brain biopsy during the second week of hospital stay.

Pathology results from microscopic examination of the brain tissue identified ‘numerous protozoan organisms’ consistent with amoebic encephalitis. The patient’s medications were re-directed to treating an amoebic abscess in the central nervous system by starting fluconazole, pentamidine, flucytosine, and clarithromycin (see Table 1 for a timeline of medications received during hospital stay). Samples from the biopsy were sent concurrently to the Centers for Disease Control (CDC).

**Table 1.** Timeline of treatment received in hospital

	Week of hospital stay												
Medication	1	2	3	4	5	6	7	8	9	10	11	12	13
Dexamethasone													
Albendazole													
Amphotericin b liposomal													
Metronidazole													
Voriconazole													
Sulfadiazine													
Fluconazole*													
Pentamidine													
Flucytosine													
Clarithromycin													
Miltefosine†													
Azithromycin*													
Tmp-Sulfa‡													

*Received additional 87 weeks.

†Received additional 3 weeks.

‡Received additional 39 weeks.

**Fig. 1.**
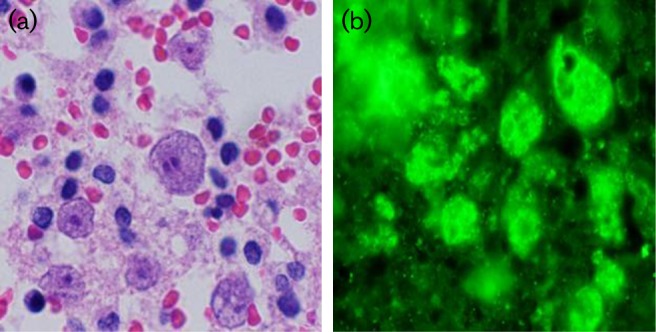
(a) Hematoxylin and eosin stain made from tissue biopsy from left frontal lobe brain biopsy. Demonstrates amoebic trophozoites under high power (×1000). These organisms have abundant amphophilic, slightly flocculent cytoplasm containing ingested cellular debris, and small eosinophilic nuclei with prominent nucleoli. (b) Indirect immunofluorescence staining at same magnification with positive fluorescent labelling for *Balamuthia* sp. (photos of CDC slides courtesy of Dr G. S. Visvesara).

Results from indirect immunofluorescence testing on paraffin-embedded tissue sections, performed at the CDC (Division of Parasitic Diseases Reference Diagnostic Laboratory), showed positive fluorescent labelling for *Balamuthia* sp. (see Fig. 1). No staining was observed for *Naegleria fowleri* or *Acanthamoeba* species. These findings supported the diagnosis of *Balamuthia mandrillaris* encephalitis. A specific *Balamuthia* polymerase chain reaction (PCR) assay was also performed at CDC to confirm the diagnosis. Samples from this biopsy specimen were later used to develop the first draft genome sequence of a strain of *Balamuthia mandrillaris* (Greninger *et al.*, 2015).

**Fig. 2.**
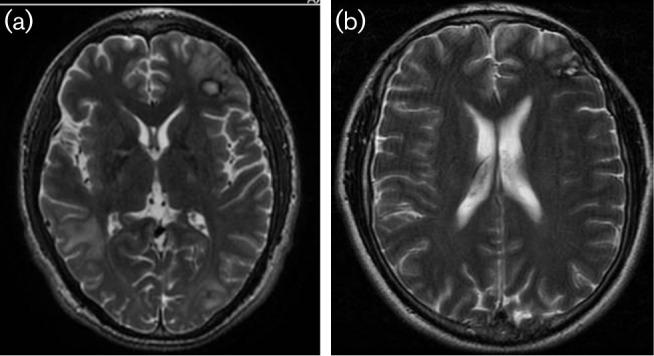
(a) HD#57 Axial T2 weighted magnetic resonance image (MRI) of brain demonstrating area of biopsy in left frontal region and oedema from mass in right temporal. Disease also noted with new satellite lesions seen in left frontal lobe and left parietal lobe. (b) MRI from two years into treatment with resolution of CNS lesions.

Within the first week of treatment, the patient developed atrial fibrillation which was also associated with prolonged QT interval. Clarithromycin was withheld because of concerns for cardiac toxicity. The patient also had mild hepatic enzyme abnormalities at the time, elevated white count, and remained symptomatic with headache and visual changes (floater and eye pain). Over the next week, he developed additional complaints of nausea, headache and itching. In addition, he developed bouts of polyuria and hypotension requiring repeated intravenous fluid boluses. A second cranial MRI was performed on hospital day (HD) 26 (see Fig. 2). The MRI was remarkable for worsening of disease in left occipital and right temporal lobe. At the time of his treatment in 2010, miltefosine was not approved by the U.S. Federal Drug Administration (FDA) for leishmaniasis or any other infection. We submitted a single patient investigational new drug application (IND) for miltefosine in this case, which was approved by the hospital’s institutional review board (IRB). The medication was started three weeks into the patient’s hospital course with one brief treatment interruption, secondary to an international shipping delay. The dose given was 150 mg oral daily. The patient continued to have several medication related side effects during the course of treatment. The main problems were intractable nausea, renal insufficiency and pancreatitis. This complicated parenteral nutritional support and medication tolerance during hospital stay. The patient’s condition declined during the first six weeks of hospital stay despite directed therapy against *Balamuthia mandrillaris*.

Fifty days into the patient’s hospital stay, the family and treating physicians considered changing treatment goals to'comfort care' due to multiple complications and the patient’s progressive clinical decline. Antibiotics were altered to parenteral administration when possible, with the exception of miltefosine, in a final effort to maximize coverage for this organism and drug penetration into the CNS.

A third MRI was performed (HD# 57) one week later after this medication switch that showed some mild interval improvement in multiple locations and showed a decrease in overall vasogenic oedema. The patient experienced gradual neurological improvement and also had resolution of hepatic, pancreatic and renal abnormalities over the following week, which coincided with treatment modification and the stopping of pentamidine. His nutritional status improved and he was able to tolerate transition of antibiotics to oral route. After almost three months in the hospital, he was discharged home. He completed approximately 114 days of miltefosine therapy with only mild nausea/vomiting as side effect.

After hospital discharge, he also continued trimethoprim-sulfamethoxazole (TMP-SMX) double strength tablet (160 mg TMP-800 mg SMX) twice daily, azithromycin 500 mg daily, and fluconazole 400 mg daily for an additional nine months until repeat MRI imaging showed continued resolution of CNS lesions. TMP-SMX was discontinued although the patient continued azithromycin and fluconazole for another year. Patient appeared to tolerate these final two medications without any problem. The brain MRI two years after presentation showed no evidence of active disease. Antibiotics were stopped at that point. Now off medication completely, the only complaints of the patient are occasional dizziness and minor visual disturbances, with no detectable neurological deficit. The patient returned to work and had a second child with his wife. 

## Discussion

The mortality rate of *Balamuthia mandrillaris* granulomatous encephalitis is very high. Of the 200 reports, only 11 survivors have been described. In a recent report in CID in 2010, Martinez *et al*. summarized seven patients who survived (Martinez *et al.*, 2010). Since that publication, an additional three survivors have been reported including a 2–year–old from Kentucky (Cary *et al*., 2010 ), an 80–year–old female from Australia (Doyle *et al*., 2011), and a 27–year–old male kidney transplant recipient ( Orozco, 2011). Of these 11 cases (see Table 2), the age range was 2 to 80 years (median =26 years) and 50 % were female. All received varied drug regiments including pentamidine (Schuster & Visvesvara, 1996), fluconazole (Doyle *et al.*, 2011), albendazole (Martinez *et al.*, 2010), itraconazole (Martinez *et al.*, 2010), macrolide (Orozco, 2011), sulfadiazine or TMP-SMX (Sanderson, 2009; Orozco, 2011) and amphotericin (Visvesvara *et al.*, 1990). Three patients also received chlorpromazine. Surgical resection of the brain mass was performed in the 10–year–old female from Peru (Seas & Bravo, 2006)and in the 80–year–old female (Doyle *et al*., 2011) whose lesion was isolated and had a complete resection. The duration of follow-up of these cases varied from three months to more than five years. In most instances an extensive neurological evaluation was not provided but most of these patients had a good outcome and were noted to be normal neurologically (e.g., some noted to have had ‘no significant neurologic sequelae’).

**Table 2.** Eleven survivors of *Balamuthia mandrillaris* since 2010

Age (Years)	Sex	Drug treatment	Duration of treatment	References
64	Male	Pentamidine 4 mg kg^−1^ daily	18 days	(Deetz, 2003)
		Trifluoperazine 20 mg daily	18 days	
		Clarithromycin 500 mg daily	24 months	
		Flucytosine 8 g daily	60 months	
		Fluconazole 400 mg daily	60 months	
		Sulfadiazine 6 g daily	60 months	
				
5	Female	Pentamidine 1 mg kg^−1^ daily	34 days	(Deetz, 2003)
		Flucytosine 110 mg kg^−1^ daily	17 months	
		Thioridazine 1 mg kg^−1^ daily	22 Months	
		Clarithromycin 14 mg kg^−1^ daily	29 months	﻿
		Fluconazole 14 mg kg^−1^ daily	29 months	
				
72	Female	Pentamidine 300 mg daily	13 days?	(Jung, 2004)
		Fluconazole 400 mg daily	Not reported	
		Sulfadiazine 1.5 gm QID	Not reported	
		Clarithromycin 500 mg tid	Not reported	
				
35	Male	Unknown	Lost to follow–up	(Centers for Disease Control and Prevention, 2008)
				
8	Male	Albendazole 400 mg daily	14 months	(Seas, 2006)
		Itraconazole 200 mg daily	14 months	
				
10	Female	Albendazole 600 mg daily	6 months	(Seas, 2006)
		Itraconazole 100 mg daily	6 months	
		Tmp-Smx (320 mg/1600 mg) daily	6 months	
				
21	Female	Itraconazole 200 mg daily	10 months	(Martinez, 2010)
		Albendazole 400 mg daily	10 months	
		Albendazole 800 mg daily	7.5 months	
		Fluconazole 450 mg daily	7.5 months	
		Ambisome 2.85 gram total	2 courses	
		Miltefosine 150 mg daily × 12, then100 mg daily	7 months	
		Tmp-Smx (640/3200) daily	45 days	
		Clarithromycin 1500 mg daily	14 days	
		Artesunate 100 mg daily	14 days	
				
80	Female	Pentamidine 300 mg daily	7 days	(Doyle, 2011)
		Liposomal amphotericin 3 mg kg^−1^ daily	21 days	
		Flucytosine 1 gm TID	7 months	
		Azithromycin 600 mg daily	7 months	
		Itraconazole 200 mg bid	7 months	
		Suphadiazine 1.5 mg QID	7 months	
				
27	Male	Sulfadiazine (dosages not reported)	126 days	(Orozco, 2011)
		Azithromycin	126 days	
		Miltefosine	126 days	
				
2	Male	Pentamidine 4 mg kg^−1^ daily	Less than 62 days	(Cary, 2010)
		Thioridazine 0.5 mg kg^−1^ daily	Less than 62 days	
		Fluconazole 150 mg kg^−1^ daily	22 months	
		Flucytosine 150 mg kg^−1^ daily	22 months	
		Sulfadiazine 200 mg kg^−1^ daily	22 months	
		Clarithromycin 14 mg kg^−1^ daily	22 months	
				
26	Male	Pentamidine 4 mg kg^−1^ daily	29days	This case
		Flucytosine 2000 mg bid	33 days	
		Sulfadiazine 1500 mg oral daily	33 days	
		Miltefosine 150 mg daily	114 days	
		Tmp-Smx 430 mg TID	1 months	
		- Reduced to 160 mg 800 mg tab oral bid	12 months	
		Fluconazole 400 mg IV q 12 & daily	25 months	
		Azithromycin 500 mg daily	25 months	

Many of the survivors initially received pentamidine. Pentamidine was originally recommended for this infection based on in vitro studies showing some activity against *Balamuthia* (Schuster & Visvesvara, 1996). However, pentamidine has known poor penetration of the blood-brain barrier into the central nervous system (7), and significant clinical toxicity associated with its use. Indeed, the six survivors that received pentamidine had reported toxicity with either hypotension, pancreatitis or severe nausea/gastrointestinal upset. In place of pentamidine, our experience suggests that miltefosine should be considered in any treatment plan. Miltefosine was originally developed as an anti-cancer drug. In recent years, it has been shown to have efficacy against leishmaniasis (Sundar, 2002). Additionally, there are in vitro data showing activity against free–living amoeba, including *Balamuthia* (Schuster *et al.*, 2006). Since this publication, miltefosine has been approved by the FDA for use in the United States for leishmaniasis. We hope that the current lack of commercial availability of the drug can be remedied that could encourage clinicians to contact the CDC to gain access to this medication for off-label use in free-living amoebic infections. Complete surgical resection is also worth considering, particularly in patients with a single brain lesion (Doyle *et al.*, 2011).

Ten of 200 reported cases survived, which is a case fatality rate of 95 %. Among the reported survivors, although many were diagnosed relatively late in their disease course,they still had a good outcome. It is possible that if cases were recognized earlier in the course of disease, an even higher proportion of cases would have improved outcomes. We hope by providing details of this case, including the protracted course and delay in clinical response improvement, clinicians will pursue medical therapy in the hope of a potential cure for this predominantly fatal disease. 

We acknowledge that the appropriate drugs, dosages, and duration of treatment remain unknown, given the rarity of the *Balamuthia mandrillaris* diagnosis. Improved diagnostic techniques including accurate PCR testing from spinal fluid and specific serologic markers by antigen or antibody detection may prove useful in earlier diagnosis without the need for invasive tissue biopsy. Ongoing research is clearly needed for the development of effective and standardized pharmacotherapy for this disease.
